# The Single-Leg-Stance Test in Parkinson’s Disease

**DOI:** 10.14740/jocmr1878w

**Published:** 2014-12-29

**Authors:** Taylor Chomiak, Fernando Vieira Pereira, Bin Hu

**Affiliations:** a Department of Clinical Neurosciences, Hotchkiss Brain Institute, Faculty of Medicine, University of Calgary, Calgary, AB, Canada

**Keywords:** Parkinson’s disease, Balance, Single, Leg, Stance, Unipedal, Test, Reliability, Bradykinesia

## Abstract

**Background:**

Timed single-leg-stance test (SLST) is widely used to assess postural control in the elderly. In Parkinson’s disease (PD), it has been shown that an SLST around 10 seconds or below may be a sensitive indicator of future falls. However, despite its role in fall risk, whether SLST times around 10 seconds marks a clinically important stage of disease progression has largely remained unexplored.

**Methods:**

A cross-sectional study where 27 people with PD were recruited and instructed to undertake timed SLST for both legs was conducted. Disease motor impairment was assessed with the Unified Parkinson’s Disease Rating Scale Part 3 (UPDRS-III).

**Results:**

This study found that: 1) the SLST in people with PD shows good test-retest reliability; 2) SLST values can be attributed to two non-overlapping clusters: a low (10.4 ± 6.3 seconds) and a high (47.6 ± 11.7 seconds) value SLST group; 3) only the low value SLST group can be considered abnormal when age-matched normative SLST data are taken into account for comparison; and 4) lower UPDRS-III motor performance, and the bradykinesia sub-score in particular, are only associated with the low SLST group.

**Conclusion:**

These results lend further support that a low SLST time around 10 seconds marks a clinically important stage of disease progression with significant worsening of postural stability in PD.

## Introduction

Parkinson’s disease (PD) and postural instability is a major clinical symptom leading to falls, injury, fear of falling and reduced quality of life [1-5]. The ability to maintain postural stability while transiently standing on a single limb is essential for normal gait and activities of daily living involving turning, stair climbing and dressing [6, 7]. As such, the time an individual can stand on one lower limb (i.e. the single-leg-stance-test (SLST) time) has been widely used as a simple assessment tool of balance in the elderly [6, 8], and in some cases in PD patients [1, 9-11]. Using force plate and posturography to measure center of pressure and moment of force signals, Adkin et al found that the Unified Parkinson’s Disease Rating Scale Part 3 (UPDRS-III) gait and posture sub-score was significantly related to the SLST [11]. Furthermore, it has been suggested that SLST times of approximately 10 s or less is associated with increased fall risk [1, 9, 10]. For example, Jacobs et al reported that an SLST cut-off of around 10 s provided the best sensitivity and specificity related to fall history in PD [1].

Bradykinesia is a cardinal clinical feature of PD which can lead to prolonged reaction times and both insufficient recruitment, and under-scaled muscle force [12]. In contrast to the gait and posture rating score of the UPDRS-III, the relationship between bradykinesia and the SLST has not been explicitly studied. It has been shown that young healthy adults are able to perform the SLST well, exhibiting long stance times that are associated with initial decreases in force variability in the stance leg [7]. However, in the elderly, this initial decrease in force variability is reduced which can result in continued higher force variability for the remaining duration of the SLST, thus leading to poorer SLST performance and reduced stance times [7]. According to this pathophysiological model, increased bradykinesia in PD may result in an inability to compensate for this increased variability and may further shorten SLST times. Thus, the current study was undertaken to address the question of whether the conventional SLST approach, which does not require special equipment (e.g. force plate), has sufficient reliability to be used in a clinical setting in revealing postural stability impairment and its association with bradykinesia in PD.

## Methods

### Subjects

Ethics approval was obtained from University Ethics Board for Human Research (REB ID: REB13-0009) and informed written consent was obtained as part of entry into the University of Calgary PD program. All (n = 27) PD subjects (n = 12 female and n = 15 male) were recruited via clinician referral by the neurologists at the Foothills Movement Disorders Clinic, and through the Parkinson’s Society of Alberta support groups. Demographic and disease information were collected for subjects with a confirmed diagnosis of PD. Subjects had a mean (standard deviation) age and disease duration of 67.1 ± 10.2 and 7.3 ± 4.2 years respectively, and an average UPDRS-III score of (16.6 ± 7.1, n = 25). The total daily dose of dopamine agonist precursor (medication) was 721 ± 301 mg (n = 19). Subjects were either in the “on” or “in between” medication state. It has been previously reported that medication state does not significantly influence the SLST [1]. This study also did not find a significant correlation between medication dose and SLST time (not shown).

### SLST

The SLST is a static balance test that has been used widely in older adults and has normative data accepted by the scientific literature [6]. Individuals were tested with eyes open; they were asked to stand on either their left or right leg and were instructed to keep their legs from touching and to maintain single-leg stance for as long as possible. A digital stopwatch was used to time as this approach has previous been shown to exhibit near perfect inter-rater reliability [6]. The test and time began once the foot was lifted off the floor, and ended when placing the lifted foot on the floor or with arm movements and the placing of their hand on a chair that was positioned beside them for support if needed. The test was terminated following a maximum of 60 s, and each leg was tested three times unless subjects performed perfectly on the first two trials. The intra-class correlation coefficient (ICC) was therefore determined based on the first two trials as these were completed by all subjects. Subjects typically alternated between legs, and subjects were allowed to rest between trials if needed. The best trial score was used for analysis which is typically used clinically [6].

### Analysis

All statistical analysis was accomplished with GraphPad Prism and SPSS software. Single measures test-retest reliability was evaluated with a two-way random ICC model with absolute agreement. Hierarchical cluster analysis using Ward’s method, based on minimizing cluster variance, was used to classify subjects based on their SLST time. This classification was then used as the dependent variable in the logistic regression models. Data are expressed as mean and standard deviation (SD) or 95% confidence interval (CI), and significance was defined as α ≤ 0.05.

## Results

### SLST test-retest reliability

This study first wanted to evaluate the trial-to-trial test-retest reliability of the SLST in people with PD. To this end, 27 people with PD were recruited and they were tested on the SLST. Based on the power of the correlation coefficient R > 0.7 as strong [13], the SLST in subjects with PD showed strong test-retest reliability with an ICC 0.82 (95% CI: 0.64 - 0.91, P < 0.01) and an ICC 0.83 (95% CI: 0.66 - 0.92, P < 0.01) for right and left legs respectively.

### SLST and the UPDRS-III

**Figure 1:**
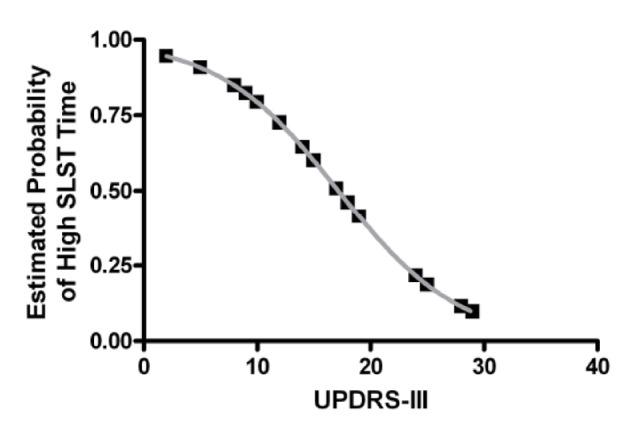
Estimated probability of having a normal SLST as a function of UPDRS-III score. Plotted is the estimated probability from the logistic regression model of being in the high SLST time group (i.e. cluster 2) as a function of UPDRS-III score. Only cluster 1, and not cluster 2, has an average SLST time that is significantly lower than that of the published age-matched normative data [6]. Thus, the probability of having a normal SLST time decreases with increasing UPDRS-III scores.

Next hierarchical cluster analysis was used to explore whether PD sub-groups may exist based on SLST times. This is important as the presence of sub-groups within a sample would contribute to large overall variability and may limit the efficacy of its use clinically to monitor disease progression [14]. To investigate this, subjects were classified via cluster analysis which found that two major PD sub-groups are clearly evident: a low SLST time group (cluster 1) and a high SLST time group (cluster 2). The low SLST time group had a mean (SD) SLST time of 10.4 ± 6.3 s while the high SLST time group had a mean (SD) SLST time of 47.6 ± 11.7 s. There was no significant difference in gender (P > 0.05; Fisher’s exact test), age (t_(25)_ = 0.87, P > 0.05; *t*-test), disease duration (t_(25)_ = 1.2, P > 0.05; *t*-test), or medication dose (t_(17)_ = -0.07, P > 0.05; *t*-test) between clusters 1 and 2, and only the low, but not the high, SLST time group had values significantly lower than that of the published age-matched normative data of 32.1 s [6] (t_(12)_ = -12.3, P < 0.05; one-sample *t*-test). However, UPDRS-III was significantly different between clusters (t_(23)_ = 2.7, P < 0.05; *t*-test; 20.3 ± 7.1 vs. 13.1 ± 5.4 for clusters 1 and 2 respectively) and was a significant predictor of cluster membership in a logistic regression model (OR = 0.83, 95% CI: 0.70 - 0.98, P < 0.05). In other words, the probability of having a high SLST time (i.e. being in cluster 2) decreases with increasing UPDRS-III score (Fig. 1).

### SLST and bradykinesia

Adkin et al previously reported that the UPDRS-III gait and posture sub-score (questions: 27 - 30) was associated with the SLST [11]. However, it has also been suggested that an initial reduced decrease of force variability may result in higher force variability during the SLST and poorer performance [7]. Thus, given that a cardinal feature of PD is bradykinesia which may result in insufficient recruitment of muscle force to compensate for this increased variability [12], this study also wanted to evaluate the UPDRS bradykinesia sub-score (UPDRS questions: 24 - 26 and 31 [15]) with respect to SLST cluster membership. Indeed, the bradykinesia sub-score was significantly associated with SLST cluster membership, correctly identifying 76% of all cases (model χ^2^_(1)_ = 7.64; P < 0.05). Consistent with Adkin et al, the gait and posture sub-score was also significantly associated with cluster membership correctly identified 64% of all cases (model χ^2^_(1)_ = 4.02; P < 0.05), although this was significantly improved with the addition of the bradykinesia sub-score to the model (68% of all cases correctly identified; Δ model χ^2^_(1)_ = 4.15; P < 0.05). Ruling-out the possibility that the association with bradykinesia was a result of hierarchical clustering, correlational analysis also revealed that the bradykinesia sub-score was significantly correlated to SLST times (Pearson r = -0.54; P < 0.05). Hence, these results suggest that the UPDRS bradykinesia sub-score is significantly associated with SLST performance in PD.

## Discussion

Balance assessment is an important tool of fall risk evaluation and a useful marker for disease staging in PD [1, 16]. The SLST is a simple balance test that has been validated and widely used, alone or as part of a larger test battery in the elderly [6, 8]. When used in PD, the SLST is usually related to fall risk, with times of approximately 10 s or less being associated with fall risk [1, 9, 10]. For example, Smithson et al found that individuals with PD with a fall history had an average SLST time of around 9.5 s [9]. Moreover, Jacobs et al reported that an SLST cut-off of around 10 s provided the best sensitivity and specificity related to fall risk [1]. Indeed, this study is consistent with these previous reports and also reports that the 10 s mark of SLST time has statistical validity that may be clinically more related to bradykinesia. While a limitation of this study is a lack of confirmed fall history, these results lend further support to the use of SLST times around 10 s as a clinically meaningful postural instability marker in PD.

Two distinct kinetic and kinematic phases have been described in performing the SLST [7]. First, during the initial dynamic phase (i.e. the first 5 s) there is a rapid decrease of force variability amplitude as subjects make postural adjustments to regain standing balance after transferring weight to one leg that occurs. During the second phase, static postural equilibrium is required to maintain balance on one foot, and it has been suggested that subjects may have difficulty maintaining balance in the static phase due to difficulty adjusting postural control during the initial phase of the SLST [6, 7]. For instance, a reduced decrease of initial force variability may result in higher force variability during the second phase of the SLST [7]. As such, people with PD may be unable to compensate for this increased variability through the insufficient recruitment and under-scaled muscle force, a principal deficit of bradykinesia [12]. In further support of this, decreased stance times for individuals may also be related to a decrease in lower extremity muscular strength and endurance as, for example, there is a positive relationship between hip flexor, extensor, and abductor strength, and SLST times [6, 17]. It is therefore not surprising that muscle weakness has also been suggested to contribute to bradykinesia in PD [12]. In addition, different levels of impairment in the neural circuitry involved in feedback modes of sensorimotor control versus supraspinal tonic drive controlled by input from the basal ganglia are also likely important [18]. As both of these possibilities are important in PD, the general balance and motor impairments associated with PD may indeed be reflected by poor SLST times. In fact, the Berg balance scale, which is scale consisting of 14 tasks common in everyday life to assess balance ability including the SLST, has also been shown to be correlated to UPDRS-III [19]. However, our results suggest that at least half of the variance associated with the complete UPDRS-III score may be related to the SLST alone, rather than the other items on the scale. For example, assuming that there are few tied ranks and the data are representative from both studies, then the results of that study indicate r^2^ is likely around 34%, which is not much larger than 26% observed in the present study based on the SLST alone.

Finally, it is noteworthy to mention that while in general there was relatively little difference between SLST times for each leg, in about a third of subjects there was quite a large difference between both legs that ranged from 11 to 55 s. Interestingly, we did not find a correlation between UPDRS-III and the poorer leg performance. It is therefore possible that in these patients the disease progression may remain relatively unilateral and may not have affected axial motor control system to the same extend as the low value SLST group. This observation also underscores the clinical importance of assessing SLST in both legs.
